# Supplementary material for: Giant Kerr response of ultrathin gold films from quantum size effect
Source: Nat Commun. 2016 Oct 10;7:13153. doi: 10.1038/ncomms13153 (PMC5062544; doi:10.1038/ncomms13153)
Supplement: Supplementary Information — Supplementary Figures 1-2, Supplementary Notes 1-8 and Supplementary References [file ncomms13153-s1.pdf]

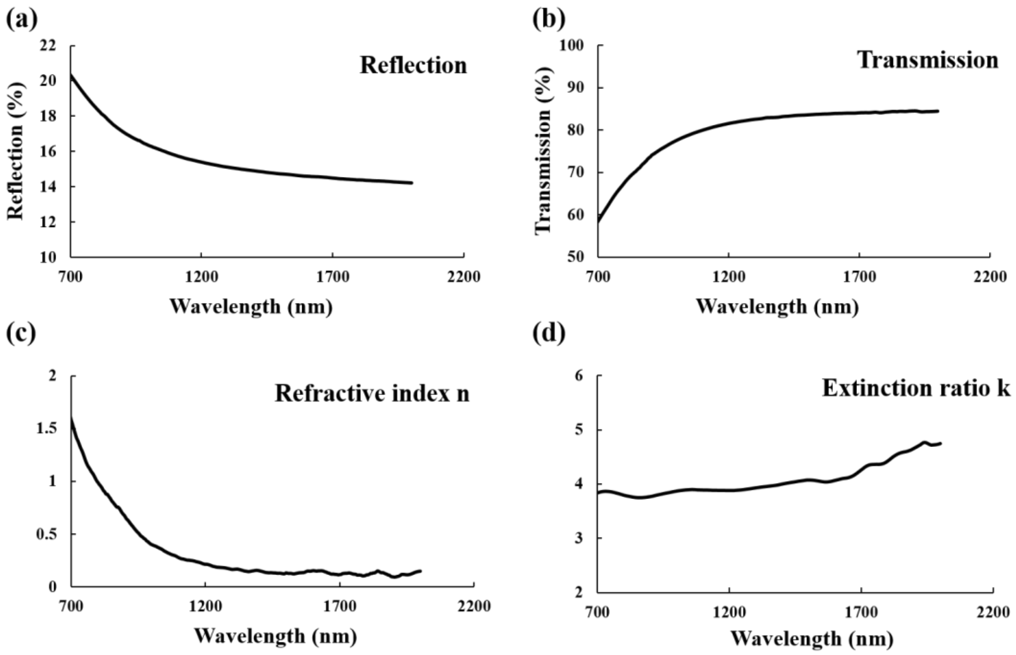

Supplementary Figure 1| Reflection and transmission measurement. (a) and (b) show the reflection and transmission curves with 45° incident angle and S-polarization for the 3 nm metal quantum well sample. The extracted *n* and *k* data are plotted in (c) and (d), respectively.

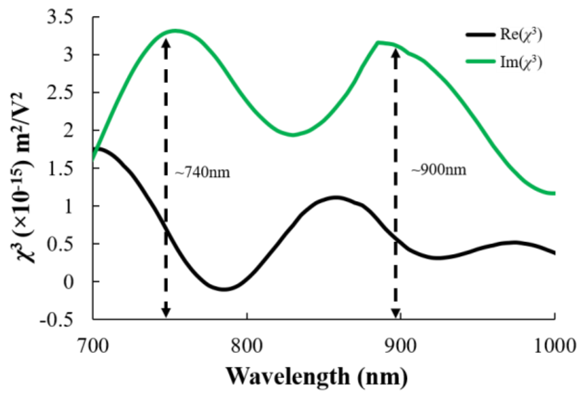

Supplementary Figure 2| Wavelength dependence of χ^3. Wavelength dependent of χ^3 for the 3 nm metallic quantum well, where real part is represented as the black line and the imaginary part as the green line.

Supplementary Note 1

Obtaining the refractive index and extinction coefficient from reflection and transmission measurements

In order to obtain the linear optical constants for the 3 nm metal quantum well, reflection and transmission (RT) measurements were first performed with different incident angles and polarizations. The measured RT curves with 45° incident angle and P- polarization for the 3nm metal quantum well sample are plotted in Supplementary Figure 1(a) and (b), respectively.

In order to extract the refractive index *n* and extinction coefficient *k* from RT curves, an extraction strategy was developed. The basic idea behind the extraction strategy is described in the following:

#1: Initial guesses n_0 and k_0 were substituted into the multilayer transfer matrix code to calculate the reflection (R_0) and transmission (T_0).

#2: Calculated R_0 and T_0 values were compared with the experimentally measured R and T. The difference f_R and f_T was used to build a Jacobian matrix for the Newton's method calculation. In order to obtain update values for *n* and *k*. The Jacobian matrix was built as follows:

$$Jb = \begin{bmatrix} -sgn(f_R) * f_{R1} & -sgn(f_R) * f_{R2} \\ -sgn(f_T) * f_{T1} & -sgn(f_T) * f_{T2} \end{bmatrix}, \tag{1}$$

where *sgn* represents the sign function. Here f_{R1}, f_{T1}, *and* f_{R2}, f_{T2} represent the R T difference between experimental value with calculated value using $n_0+\Delta n$, k_0, and n_0, $k_0+\Delta k$, respectively. The new *n*, *k* value was updated with

$$\begin{bmatrix} n_{new} \\ k_{new} \end{bmatrix} = Jb \backslash \begin{bmatrix} f_R \\ f_T \end{bmatrix}. \tag{2}$$

#3: The updated *n* and *k* were substituted back into the multilayer transfer matrix code to calculate the updated RT.

These processes were repeated until a final *n* and *k* was obtained such that differences between the calculated and experimentally measured RT values were smaller than a pre-defined threshold value. The major feature of the strategy is that it considers *n* and *k* as independent variables, and performs a two-dimensional Newton's method. Such a strategy enables a quick and efficient finding of *n* and *k* values corresponding to the experimental RT data.

Supplementary Note 2

Nonlinear optical response of a metal quantum well

A Quantum Electrostatic Model [1] (QEM) can be used to describe the electron dynamics within thin metal films. This method utilizes an iteration algorithm between Schrödinger

equation and Poisson equation to identify the electron density distribution, eigen-states and wave functions. Once the self-consistent eigen-states and wave functions are obtained, the optical properties (both linear and nonlinear) can be deduced. Here, a density matrix formulation is chosen to derive the nonlinear optical susceptibilities of a metal quantum well following the perturbation theory. The details of such a derivation can be found in Ref 5, where the main steps are summarized as follows.

The density matrix is a mathematical tool for describing a quantum system when it is in a mixed state [2]. The corresponding density operator is expressed as $\hat{\rho} = \sum_s p_s |\psi_s >< \psi_s|$, where p_s is the probability that the system is in state $|\psi_s >$ such that $\sum_s p_s = 1$. By substituting the wave function represented by density matrix into Schrödinger equation, the time evolution equation of the density matrix is obtained as

$$\frac{d\rho_{nm}}{dt} = -i\omega_{nm}\rho_{nm} - \frac{i}{\hbar}[\hat{V},\hat{\rho}]_{nm} - \gamma_{nm}(\rho_{nm} - \rho_{nm}^{(eq)}) , \quad (3)$$

where $\omega_{nm} = (E_n - E_m)/\hbar$ is the transition frequency between eigen-energies, the commutator $[\hat{V},\hat{\rho}] = \hat{V}\hat{\rho} - \hat{\rho}\hat{V}$, γ_{nm} is the damping rate and $\rho_{nm}^{(eq)}$ is the equilibrium value. The last term on the right-hand side is included phenomenologically for the relaxation process. Here the Hamiltonian is separated into two parts: $\hat{H} = \hat{H}_0 + \hat{V}(t)$, where the first term represents the Hamiltonian of a free atom and the second term represents the interaction of the atom with the external radiation field (assumed to be a small term). In the electric dipole approximation, $\hat{V}(t) = -\hat{\mu} \cdot E(t)$, where $\hat{\mu} = -e\hat{r}$ is the dipole moment operator.

In most of the cases, Supplementary Equation (3) cannot be solved exactly. Following the standard perturbation theory, all the high order (M_{th} order) corrections to the steady-state solution $\rho_{nm}^{(0)} = \rho_{nm}^{(eq)}$ can be found through the relation

$$\rho_{nm}^{(M)} = \int_{-\infty}^{t} \frac{-i}{\hbar}[\hat{V}(t'),\hat{\rho}^{(M-1)}]_{nm} e^{(i\omega_{nm}+\gamma_{nm})(t'-t)} dt' . \quad (4)$$

By relating the expectation value of the induced dipole moment $< \hat{\boldsymbol{\mu}}(\omega_1 + \omega_2 + \omega_3) > = \sum_{nm} \rho_{nm}^{(3)} \boldsymbol{\mu}_{mn}$ with the definition of nonlinear polarization $P_k(\omega_1 + \omega_2 + \omega_3) = \varepsilon_0 \sum_{jih} \sum_{123} \chi_{kjih}^{(3)}(\omega_1 + \omega_2 + \omega_3; \omega_1, \omega_2, \omega_3)\, E_j(\omega_1)E_i(\omega_2)E_h(\omega_3)$, one can obtain the general expression for the third order nonlinear susceptibilities for a metal quantum well as

$$\chi_{kjih}^{(3)}(\omega_1 + \omega_2 + \omega_3; \omega_1, \omega_2, \omega_3) = \frac{N}{\varepsilon_0 \hbar^3} P_I \sum_{nmpl} *$$

$$\Bigg\{ \frac{\left[\rho_{mm}^{(0)} - \rho_{ll}^{(0)}\right] \mu_{mn}^{k} \mu_{np}^{j} \mu_{pl}^{i} \mu_{lm}^{h}}{[\omega_{nm} - \omega_1 - \omega_2 - \omega_3 - i\gamma_{nm}]\left[\omega_{pm} - \omega_1 - \omega_2 - i\gamma_{pm}\right][\omega_{lm} - \omega_1 - i\gamma_{lm}]}$$

$$- \frac{\left[\rho_{ll}^{(0)} - \rho_{pp}^{(0)}\right] \mu_{mn}^{k} \mu_{np}^{j} \mu_{lm}^{i} \mu_{pl}^{h}}{[\omega_{nm} - \omega_1 - \omega_2 - \omega_3 - i\gamma_{nm}]\left[\omega_{pm} - \omega_1 - \omega_2 - i\gamma_{pm}\right]\left[\omega_{pl} - \omega_1 - i\gamma_{pl}\right]}$$

$$+\frac{\left[\rho_{ll}^{(0)}-\rho_{nn}^{(0)}\right]\mu_{mn}^{k}\mu_{pm}^{j}\mu_{lp}^{i}\mu_{nl}^{h}}{[\omega_{nm}-\omega_1-\omega_2-\omega_3-i\gamma_{nm}]\left[\omega_{np}-\omega_1-\omega_2-i\gamma_{np}\right][\omega_{nl}-\omega_1-i\gamma_{nl}]}$$

$$\left.-\frac{\left[\rho_{pp}^{(0)}-\rho_{ll}^{(0)}\right]\mu_{mn}^{k}\mu_{pm}^{j}\mu_{nl}^{i}\mu_{lp}^{h}}{[\omega_{nm}-\omega_1-\omega_2-\omega_3-i\gamma_{nm}]\left[\omega_{np}-\omega_1-\omega_2-i\gamma_{np}\right]\left[\omega_{lp}-\omega_1-i\gamma_{lp}\right]}\right\} \tag{5}$$

where N is the density of free electrons, $\rho_{ll}^{(0)}$ is the population of electrons in state l, and $\boldsymbol{\mu}_{mn} = -e < m|\hat{\mathbf{r}}|n >$ is dipole transition element related to the transition between state m and n. The intrinsic permutation operator P_I considers all possible permutation. In the special case of Kerr response, three different permutations need to be considered: (1) $\omega_1 = \omega_2 = \omega_0, \omega_3 = -\omega_0$; (2) $\omega_1 = \omega_3 = \omega_0, \omega_2 = -\omega_0$; and (3) $\omega_2 = \omega_3 = \omega_0, \omega_1 = -\omega_0$.

As can be seen from Supplementary Equation (5), the nonlinear optical response of a quantum material system depends only on a number of limited parameters, such as the transition matrix elements μ_{nm} and density of states N, and the damping rate γ_{nm}. When compared to traditional nonlinear crystals, the advantages of a metal quantum well is the large transition matrix elements μ_{nm}. For traditional nonlinear crystals, the nonlinear response comes from the bounded electrons, which are confined within molecular length. The transition happens between the energy states within the molecular size [3] and μ_{nm} is usually on the level of few e·pm. For metal quantum well systems, the nonlinear response comes from the quantized free electrons, which are confined within the quantum well. The transition happens between the quantized sub-bands, where their wave functions in the quantum well direction have an extension that is comparable to the well width, and μ_{nm} is usually on the level of few e·nm, which is much larger than that for traditional nonlinear crystals. This number is also about 1 order of magnitude larger than interband transition dipole moment of bulk gold [4]. Although semiconductor quantum well has similar μ_{nm}, but its free electron density [3] is typically on the 10^{23} m^{-3} level, much lower than that of metal quantum well. Therefore, one can easily see that by having large transition matrix element and free electron density at the same time, metal quantum well systems is among the most promising material systems for nonlinear applications.

Supplementary Note 3

Extraction of the Kerr coefficient n_2 from nonlinear spectrum broadening measurement

As is known from nonlinear optics theory, the spectrum of an optical pulse would be broadened as it propagates through a Kerr medium due to the intensity-dependent refractive index, which is also called self-phase modulation (SPM). The SPM-induced spectral broadening is proportional to the nonlinear phase Re$(n_2)\times I\times k\times L_{eff}$, where I is the light intensity, k is the wave vector and L_{eff} is the effective length [5]. Therefore, by measuring the nonlinear spectral broadening, one can extract the Kerr coefficient as well. Full simulation of optical pulse propagation is performed, and through comparing the calculated spectrum with the experimental

result, the real part of Kerr coefficient Re(n_2) of 3.82×10^{-9} cm^2 W^{-1} is obtained for 900 nm wavelength.

Note that, the imaginary part of Kerr coefficient would lead to the amplitude changes of the pulse, which would also lead to the change in the pulse spectrum. In general, this effect also needs to be considered, especially for the case of a strong nonlinear absorption. In our case, from the shape of the experimental measured transmitted spectrum, we find that the impact of nonlinear absorption is negligible, which agree with the *z*-scan measurement result. Using the Kerr coefficient of n_2=3.82×10^{-9} cm^2 W^{-1}, a $|\chi^{(3)}|$ of 2.01×10^{-15} m^2 V^{-2} is obtained for the 3 nm gold quantum well at 900 nm, which is quite close to the value obtained from the *z*-scan measurement.

Supplementary Note 4

Discussion of the resonant transition for the quantum well states

Using Supplementary Equation (5) and considering thickness variation induced resonance broadening, one can plot the wavelength dependent $\chi^{(3)}$ as shown in the Supplementary Figure 2.

As can be seen from the Supplementary Figure 2, the $\chi^{(3)}$ shows two resonant transitions around the wavelength of 740 nm and 900 nm, which indeed align with the peak of the imaginary part of $\chi^{(3)}$. This behavior is similar to the Lorentzian resonance of linear susceptibility where its imaginary part aligns with the resonance transition peak. However, from the above $\chi^{(3)}$ expression, the third order nonlinear susceptibility is not a simple Lorentzian oscillator model. There are multiple oscillation terms presented in the denominator. Also, there exist multiple quantum energy levels so that each resonance will be affected by the nearby resonances. Therefore, the overall wavelength dependence $\chi^{(3)}$ won't be a perfect Lorentzian profile.

The quantity that has direct physical meaning is the Kerr coefficient, which can be directly linked to the intensity-dependent refractive index and loss. The relation between Kerr coefficient n_2 and $\chi^{(3)}$ is

$$\mathrm{Re}\{n_2\} = 283\frac{\mathrm{Im}\{\chi^{(3)}\}\times k + \mathrm{Re}\{\chi^{(3)}\}\times n}{n\left(n^2+k^2\right)}$$

$$\mathrm{Im}\{n_2\} = 283\frac{\mathrm{Im}\{\chi^{(3)}\}\times n - \mathrm{Re}\{\chi^{(3)}\}\times k}{n\left(n^2+k^2\right)} \tag{6}$$

As shown in Fig. S1, the extinction coefficient *k* is much larger than the refractive index *n*. From the above equation, one can see that the peak location of the real part of the n_2 will be mainly determined by the peak of the imaginary part of the $\chi^{(3)}$. As a result, the peak of the real part of n_2 is closer to the resonant transition, as shown in Fig. 3 in the manuscript. While the Kerr

coefficient can be directly linked to the nonlinear performance, the third order susceptibility gives more clear physical picture regarding the resonant transition.

Compared with the intrinsic bulky $\chi^{(3)}$ value listed in Table I, since for metal, *n* is usually smaller than *k*, and in our metallic quantum well, the Re(n_2) is much larger than Im(n_2), so the imaginary part of $\chi^{(3)}$ will dominate. However for typical bulky gold, usually the nonlinear absorption (Im(n_2)) is dominated, so the result of $\chi^{(3)}$ can be different with the one from the metallic quantum well.

Supplementary Note 5

Impact of thickness variation

Supplementary Equation (5) assumes a perfect flat quantum well. In reality, there would be thickness variations, as the gold quantum well sample shown in Fig. 1 (b). Such thickness variation would lead to the smooth of resonance features of the material response. To include this effect, a Gaussian broadening approach is adopted [6]. More specifically, the final nonlinear response is obtained through:

$$\mathrm{X}^{(3)}(\omega) = \sum_{nm} \int_{-\infty}^{\infty} \Upsilon(\Delta\omega_{nm}, \omega_{nm}) \chi^{(3)}(\omega) d\omega. \tag{7}$$

where $\Upsilon(\Delta\omega_{nm}, \omega_{nm}) = \frac{1}{\Delta\omega_{nm}\sqrt{2\pi}} e^{-\frac{(\omega-\omega_{nm})^2}{\Delta\omega_{nm}^2}}$ is the broadening function for a resonant transition between state m and n centred at frequency ω_{nm}. For a metal quantum well, the eigen-energy for state n can be approximated as $E_n \approx \frac{(n\pi\hbar)^2}{2md^2}$, where m is the electron effective mass, and d is the thickness of the quantum well. Therefore, a thickness variation of Δd would lead to a variation in the eigen-energy as $\Delta E_n = -\frac{(n\pi\hbar)^2}{2md^2} 2\frac{\Delta d}{d} = -2\frac{\Delta d}{d} E_n$. The thickness variation obtained from the AFM measurement data shown in Fig. 1(c) leads to a broadening factor $\hbar\Delta\omega_{nm}$ of 0.123 eV and 0.182 eV for the transitions between 6th to 5th and 7th to 6th eigen-states.

The fluctuation in the thickness would lead to the broadening of the transition resonances, which would contribute to the experimental result of a relatively large numbers for n_2 or $\chi^{(3)}$ away from the resonance. Note that the thickness variation is not the only reason for a broad resonance for the nonlinear response. The relative small dipole dephasing time of gold here also contributes to this effect [7].

Supplementary Note 6

Discussion of the out-of-equilibrium electron distribution on the Kerr nonlinearity

Due to the absorption of incident laser power, electrons would be driven out-of-equilibrium, and they are termed as hot electrons. The typical time for out-of-equilibrium hot

electron gas to reach internal thermal equilibrium ranges from 0.1 to 1 *ps*. This process is termed as athermal regime[8]. After the athermal region, hot electrons experience typical thermal effect and transfer energy to the lattice. In this section, we first discuss the impact of such athermal effect (electron-redistribution) on the Kerr nonlinearity. Considering the fact that the incident photons have energy much less than the interband transition of gold, a light pulse will mostly induce intraband transitions within the conduction band. In a metal quantum well system, the transition of electrons between quantized energy states leads to population redistribution of each quantized states in the conduction band. In this section, we show that the impact of such electron distribution changes to the Kerr nonlinear response in our measurement can be safely neglected.

Direct support comes from the fact that the measured nonlinear spectral broadening is almost symmetric. The reason is as follows: Optical Kerr nonlinearity is an instantaneous response. An optical pulse sees a nonlinear phase shift profile exactly following the shape of the pulse itself inside a Kerr medium. For an input of symmetric Gaussian pulse, the change in the refractive index felt by the pulse itself is also symmetric Gaussian shape. As a consequence, the laser pulse would develop a spectral broadening that is symmetric.

The photon-induced athermal electron re-distribution is not an instantaneous process as Kerr effect. The excitation process of an electron by absorbing an incident photon can be viewed as an instantaneous process. However, the relaxation process is non-instantaneous. The typical relaxation time of noble metal quantum well studied from photon-emission measurement [9, 10] ranges from 5 to 10 fs. Therefore, an optical pulse would not experience a symmetric refractive index change in temporal domain. As a consequence, the pulse spectrum would not develop a symmetric broadening in this case.

To further rule out other possibilities, we also have performed the spectral broadening experiment on 3 nm Au with Si substrate and no spectrum broadening is found in this case. The quantum confinement of the free electrons in the gold film comes from the two barriers on both sides. Silicon is a semiconductor with a small band gap (less than 1.2 eV), while quartz is a dielectric with a much larger bandgap (around 8.9 eV). Therefore, the confinement effect from a Si substrate is much weaker than a quartz substrate's, and hence with a much lower nonlinear response that comes from the quantum confinement effect.

So one can safely justify the fact that the contribution of this out-of-equilibrium electron distribution can be neglected compared with the larger Kerr response from quantum size effect.

Supplementary Note 7

Discussion on thermal effects

Thermal effect can play a very important role in *z*-scan experiment [11], and the thermal effective "third" order nonlinearity in metals could be larger than that from most of the nonlinear crystals [7]. It has been reported that most of the measured *z*-scan signal can come from thermal effect in some cases. This section puts more discussion on thermal effects. Two kinds of thermal effect need to be considered in the case of using pulse laser with certain pulse width and

repetition rate. The first one is the thermal effect within a single pulse envelope where the thermal effect is induced by the laser pulse itself when the hot electron relaxed through electron-electron and electron-phonon interaction. The other effect is the accumulated thermal effect, which becomes critical for a pulsed laser with high repetition rate. For the first case, as shown by previous pump-probe experiments [12] and the two temperature model [13], the typical hot-electron delay time for gold is about 500 fs, which is much longer than the laser pulse width (80 fs) used in this study. Therefore, the impact of hot electron effect can be safely neglected in our experiment.

The second source of thermal contribution comes from accumulated effect from the pulse train, which can be ruled out by the spectral broadening measurement. The difference in *z*-scan and spectral broadening measurement is that the former measures the spatial intensity dependence of the refractive index, while the latter measures the temporal intensity dependence of refractive index. In the case of *z*-scan, the accumulated thermal effect may lead to a spatially-dependent refractive index change because of the invariant laser intensity profile. Therefore, this type of thermal effects (which mainly comes from the contribution of the repetitive pulse train) can express similar instantaneous Kerr response (which only comes from the single pulse itself). While a stabilized accumulated thermal effect due to the repetitive pulse train is constant in the temporal domain and is independent on the each pulse shape. In this case, the accumulated thermal effect cannot contribute to the spectral broadening.

To further confirm that the measured signal is from Kerr nonlinearity, the self-phase modulation induced spectral broadening is performed. The pulse spectrum broadening experiment results (shown in Figure 4) show that the third order nonlinear coefficient measured from *z*-scan experiment comes from Kerr contribution and the thermal contribution is quite small, almost negligible on the power level chosen in this study.

Supplementary Note 8

Comparison of the nonlinear performance of gold quantum well with plasmonic gold nano particles

In this section, we provide a rough estimation regarding the nonlinear performance of quantum wells and plasmonic nanoparticles by using the same amount of gold. Let's consider the following two cases.

Case (1): Plasmonic nanoparticles. We consider a dielectric medium of size L×L×L with embedded gold nano spheres. Assume the volume fraction of gold nano sphere is p, the effective third order susceptibility can be obtained from the Maxwell-Garnett theory[14] as $\chi_{eff}^{(3)} = p|f|^2 f^2 \chi_m^{(3)}$, where $\chi_m^{(3)}$ is the intrinsic value for bulk gold and f is the field enhancement factor from localized surface plasmon resonance (LSPR). The Kerr phase accumulation for such a medium is proportional to : $\chi_{eff}^{(3)} L = p|f|^2 f^2 \chi_m^{(3)} L$.

Case (2): Quantum wells. With the same transverse plane geometry (L×L), it corresponds to multiple quantum wells with total thickness of pL. The corresponding Kerr phase accumulation

can be written as: $\chi_{qw}^{(3)} pL = 10^4 p \chi_m^{(3)} L$, considering that the nonlinear susceptibility of gold quantum well is 4 orders larger than that of bulk gold.

In other nonlinear effects, such as degenerated four wave mixing or third harmonic generation, the nonlinear performance now is proportional to $\left|\chi_{eff}^{(3)} L\right|^2 = p^2 |f|^8 \left|\chi_m^{(3)}\right|^2 L^2$ for the composite medium[15], i.e. case (1) and $\left|\chi_{qw}^{(3)} pL\right|^2 = 10^8 p^2 \left|\chi_m^{(3)}\right|^2 L^2$ for the metallic quantum well, i.e. case (2).

One can see that the performance of the plasmonic particles mainly depends on the local field enhancement factor *f*. If *f*=10, the two cases have similar performance. According to the reference[14], f for gold is typically less than 10, which indicates that metal quantum well approaches very likely possesses better nonlinear performance. For coupled nano structures or other plasmonic particles with sharp edges, the maximum local field enhancement factor can be locally much larger[16]. But in this case, the whole nano structure cannot be treated as a uniform medium with electric field varies across the particle. In this case, the above theory does not apply, and one need to integrate the whole nano structure to evaluate the overall nonlinear performance.

In addition, there are additional unique advantages for the metallic quantum wells:

(a) The high $\chi_m^{(3)}$ in quantum well system is intrinsically a broadband phenomena as demonstrated in our experiments. On the contrary, the field enhancement from LSPRs is usually a narrow band effect. Higher field enhancement is typically companied by narrower bandwidth.

(b) Metallic quantum well is a uniform medium, which is free of the scattering effect. Therefore, the metallic quantum well is a better option for specific integrated applications where scattering is not allowed, especially for the case where large nano particles are used and scattering can not be neglected.

(c) Metallic quantum well can also be combined with plasmonic nanostructures for better performance. For example, one can put plasmonic resonators close to quantum wells, so that the merits from both cases can be married.

Supplementary References

1. Qian, H., Xiao, Y., Lepage, D., Chen, L., Liu, Z. Quantum Electrostatic Model for Optical Properties of Nanoscale Gold Films. *Nanophotonics* **4**, 413-418 (2015).

2. Fano, U. Description of States in Quantum Mechanics by Density Matrix and Operator Techniques. *Rev. Mod. Phys.* **29**, 74-93 (1957).

3. Schmittrink, S., Chemla, D. S., Miller, D. A. B. Linear and Nonlinear Optical-Properties of Semiconductor Quantum Wells. *Adv. Phys.* **38**, 89-188 (1989).

4. Hache, F., Ricard, D., Flytzanis, C., Kreibig, U. The optical kerr effect in small metal particles and metal colloids: The case of gold. *Applied Physics A* **47**, 347-357.

5. Agrawal, G. P. *Nonlinear fiber optics*. Academic press (2007).

6. Hamedi, H. R., Juzeliūnas, G. Phase-sensitive Kerr nonlinearity for closed-loop quantum systems. *Phys. Rev. A* **91**, 053823 (2015).

7. Boyd, R. W. Nonlinear Optics, 3rd Edition. *Nonlinear Optics, 3rd Edition*, 1-613 (2008).

8. Guillet, Y., Rashidi-Huyeh, M., Palpant, B. Influence of laser pulse characteristics on the hot electron contribution to the third-order nonlinear optical response of gold nanoparticles. *Phys. Rev. B.* **79**, 045410 (2009).

9. Paggel, J., Miller, T., Chiang, T.-C. Quantum-well states as Fabry-Pérot modes in a thin-film electron interferometer. *Science* **283**, 1709-1711 (1999).

10. Chiang, T.-C. Photoemission studies of quantum well states in thin films. *Surface Science Reports* **39**, 181-235 (2000).

11. de Nalda, R., *et al.* Limits to the determination of the nonlinear refractive index by the Z-scan method. *J. Opt. Soc. Am. B.* **19**, 289-296 (2002).

12. Sun, C. K., Vallée, F., Acioli, L. H., Ippen, E. P., Fujimoto, J. G. Femtosecond-tunable measurement of electron thermalization in gold. *Phys. Rev. B.* **50**, 15337-15348 (1994).

13. Conforti, M., Della Valle, G. Derivation of third-order nonlinear susceptibility of thin metal films as a delayed optical response. *Phys. Rev. B.* **85**, 245423 (2012).

14. Papadopoulos, M. G., Sadlej, A. J., Leszczynski, J. *Non-linear optical properties of matter*. Springer (2006).

15. Ricard, D., Roussignol, P., Flytzanis, C. Surface-mediated enhancement of optical phase conjugation in metal colloids. *Opt. Lett.* **10**, 511-513 (1985).

16. Hao, E., Schatz, G. C. Electromagnetic fields around silver nanoparticles and dimers. *The Journal of chemical physics* **120**, 357-366 (2004).
